# Extracellular Vesicles Modulate Host-Microbe Responses by Altering TLR2 Activity and Phagocytosis

**DOI:** 10.1371/journal.pone.0089121

**Published:** 2014-02-20

**Authors:** Jeroen van Bergenhenegouwen, Aletta D. Kraneveld, Lieke Rutten, Nienke Kettelarij, Johan Garssen, Arjan P. Vos

**Affiliations:** 1 Nutricia Research, Utrecht, The Netherlands; 2 Department of Pharmacology, Utrecht Institute for Pharmaceutical Sciences, Utrecht University, Utrecht, The Netherlands; University of California Merced, United States of America

## Abstract

Oral delivery of Gram positive bacteria, often derived from the genera Lactobacillus or Bifidobacterium, can modulate immune function. Although the exact mechanisms remain unclear, immunomodulatory effects may be elicited through the direct interaction of these bacteria with the intestinal epithelium or resident dendritic cell (DC) populations. We analyzed the immune activation properties of Lactobacilli and Bifidobacterium species and made the surprising observation that cellular responses in vitro were differentially influenced by the presence of serum, specifically the extracellular vesicle (EV) fraction. In contrast to the tested Lactobacilli species, tested Bifidobacterium species induce TLR2/6 activity which is inhibited by the presence of EVs. Using specific TLR ligands, EVs were found to enhance cellular TLR2/1 and TLR4 responses while TLR2/6 responses were suppressed. No effect could be observed on cellular TLR5 responses. We determined that EVs play a role in bacterial aggregation, suggesting that EVs interact with bacterial surfaces. EVs were found to slightly enhance DC phagocytosis of *Bifidobacterium breve* whereas phagocytosis of *Lactobacillus rhamnosus* was virtually absent upon serum EV depletion. DC uptake of a non-microbial substance (dextran) was not affected by the different serum fractions suggesting that EVs do not interfere with DC phagocytic capacity but rather modify the DC-microbe interaction. Depending on the microbe, combined effects of EVs on TLR activity and phagocytosis result in a differential proinflammatory DC cytokine release. Overall, these data suggest that EVs play a yet unrecognized role in host-microbe responses, not by interfering in recipient cellular responses but via attachment to, or scavenging of, microbe-associated molecular patterns. EVs can be found in any tissue or bodily fluid, therefore insights into EV-microbe interactions are important in understanding the mechanism of action of potential probiotics and gut immune homeostasis.

## Introduction

Mammals live in symbiosis with commensal bacteria and it has long been recognized that host-bacterial associations yield mutual benefits [1]. Over the last years it has become clear that the microbiota play a critical role in the maintenance of gut homeostasis and immunological tolerance [2]. Accumulating evidence suggests that changes in the gut microbiota composition occur and may be causally involved in an array of immunopathologies such as inflammatory bowel diseases (IBD) and systemic immune diseases such as rheumatoid arthritis, type 1 diabetes and allergic diseases [3]. Strategies aimed at influencing dysbiosis via the oral administration of lactic acid bacteria (LAB), such as strains from the *Bifidobacterium* and *Lactobacillus* genera, have been proven beneficial in IBD [4] and atopic diseases [5]. Although their exact mechanism of action still remains obscure [6], LAB administration has been shown to enhance the barrier function of intestinal epithelial cells (IEC)[7] and to induce regulatory T-cells both systemically [8] and within the large intestine [9], most likely via ligation of specific Toll-like receptors (TLR) [10]. IEC form a barrier separating commensal bacteria and host connective tissue. Next to their barrier function, recognition of bacteria by IECs contributes to gut immune homeostasis via the release of soluble factors that regulate immune-cell function [11]. Dendritic cells (DC) residing in the lamina propria sample the microbiota by extending dendrites between IECs into the lumen [12] and interact directly with bacteria passing through M-cells, a special subset of IEC found within Peyer’s patches [13]. DCs express a full complement of pattern recognition receptors (PRR) that allows for the direct recognition and activation by microbes. As DCs are potent stimulators of naïve T-cells, microbial induced DC activation in concert with IEC soluble factors shape T-cell polarization towards effector and regulatory populations [11], [14]. TLRs are part of the larger family of PRRs and are best known for their involvement in the recognition of microbe-associated molecular patterns (MAMP) [15]. Depending on the type, species or strain of bacteria, it carries ligands for various TLRs on its surface [16]. LAB cell wall constituents typically include lipoteichoic acids (LTA), peptidoglycan (PG) and lipoproteins (LP), which are all able to interact with TLR2 [17].

Extracellular vesicles (EV) recently have gained scientists’ interest due to their role in cell to cell communication locally or at a distance. EVs typically range in diameter from 30 nm to 1 µm and can be regarded as cargo containers used to exchange biomolecules and genetic information able to both activate or inhibit recipient cells [18], [19]. The term EVs encompasses all types of secreted vesicles including exosomes, microvesicles and ectosomes [20] which originate from a broad range of cell types, including IEC [21] and dendritic cells [22]. Currently, there is no information available on the half-life of EVs, but EVs originating from epithelial, tumor and hematopoietic cells have been isolated from bodily fluids such as human plasma [23], serum [24], bronchoalveolar lavage fluid [25] and milk [26]. This suggests that EV form stable structures able to convey their information over long distances, playing a role in normal physiology and disease pathogenesis [27]. Immunosuppressive effects of EVs collected from different body fluids have been reported. For instance, EVs isolated from breast milk have been found to inhibit IFNγ production by activated PBMCs and by increasing the number of regulatory T-cells [26]. Additionally, placenta-derived EVs and EVs isolated from the maternal peripheral circulation have been found to modulate T-cell function, possibly attenuating immune responses to the fetus [28]. EVs are also reported to play a role in immunological tolerance. IEC derived EVs, isolated from serum after antigen feeding, are capable of inducing antigen specific tolerance [21]. Moreover, EVs released from intestinal mucosal cells suppress activation of liver NKT cells via transport of mucus-derived PGE_2_, potentially identifying a pathway for induction of immune tolerance towards intestinal-related antigens [29]. Based on the described immunosuppressive and tolerance inducing effects of EVs we hypothesize that EVs in intestinal tissue play a role in the maintenance of intestinal homeostasis by modulating host-microbe responses. Until now, EVs were thought to modify the cellular responses of the recipient cells. Our observations in this study suggest that EVs play a role in host-microbe responses mainly through their interaction with specific MAMPs or LAB preventing or enhancing subsequent cellular responses.

## Materials and Methods

### Bacterial Fermentation and Enumeration

*Lactobacillus* strains (*L. rhamnosus* NutRes 1, *L. plantarum* NutRes 8 and *L. caseï* CNCM I-1518) and *Bifidobacterium* strains (*B. longum* NutRes 266, *B. breve* NutRes 200 and *B. animalis* DN173010) were grown at 37°C in a 400 ml reactor containing MRS broth (Oxoid, Badhoevedorp, The Netherlands) supplemented with 0.5 g/l L-cysteine for Bifidobacteria. The pH was maintained at 6.5 by addition of NaOH. To ensure anaerobic conditions the headspace was flushed with N_2_ or a gas mixture consisting of 5% H_2_, 5% CO_2_ and 90% N_2_ for Bifidobacteria. Bacteria were harvested in the early stationary phase, washed in PBS and stored with glycerol 20% (w/v), in aliquots at −80°C. Cell counts were determined by plating serial dilutions (CFU) and fluorescent microscopy by staining with DAPI.

### Cell Lines and Reagents

Ultrapure lipopolysaccharide (LPS) derived from E. coli K12 and flagellin, FLA-ST (both from Invivogen, Toulouse, France) were used at the indicated concentrations. Synthetic bacterial lipopeptides Pam_3_CSK_4_, Pam_2_CKS_4_, FSL-1 (all from EMC microcollections, Tübingen, Germany) and purified LTA from *S. aureus* (Invivogen, Toulouse, France) were used at the indicated concentrations. Monocyte cell line THP-1 Blue-CD14 containing the NFκB reporter pNiFty2-SEAP, HEK293 TLR2-TLR6 stable transfectants and HEK293 TLR null control cells were purchased from Invivogen, Toulouse, France. HEK293 TLR2-TLR6 transfectants were stably transfected with the NFκB reporter plasmid pNiFty2-Luc (Invivogen, Toulouse, France). Cells were maintained in RPMI-1640 medium supplemented 10% FBS and the appropriate antibiotics according to the manufacturer’s protocols.

### Serum Fractionation

Mouse sera collected from TLR2 knockout mice and wild types were generously provided by Shahla Abdollahi-Roodsaz (Radboud UMC, Nijmegen, The Netherlands). Human plasma was obtained after buffy coat separation using Leucosep tubes as described in the section on the differentiation of dendritic cells. Heat-inactivated human serum AB and fetal bovine serum were purchased from Lonza, Verviers, Belgium and Fisher Scientific, Landsmeer, The Netherlands respectively. 0.22 µm filtered serum was mixed with ExoQuick Exosome Precipitation Solution (System Biosciences) at a ratio of 4∶1 (serum: ExoQuick) and incubated at 4°C for 30mins and subsequently spun down. Depleted serum was transferred to a new tube. The EV-rich pellet was carefully rinsed with PBS and reconstituted in the original volume with the appropriate medium. Serum fractions were used 5% (v:v) or at the indicated concentrations.

### Differentiation of Dendritic Cells

Human primary peripheral blood mononuclear cells (PBMCs) were isolated from buffy coats obtained from healthy blood donors at the Sanquin Bloodbank, Nijmegen, The Netherlands. The mononuclear cell fraction was obtained by density centrifugation of blood diluted 1∶1 in PBS using Leucosep tubes (Greiner, Alphen a/d rijn, The Netherlands) according to the manufacturer’s instructions. CD14 MACS isolation beads were used to positively select monocytes. Monocytes were cultured in RPMI-1640 medium supplemented with 10% FBS, 10 ng/ml IL-4 and 5 ng/ml GM-CSF (both cytokines and magnetic beads obtained from Miltenyi Biotec, Leiden, The Netherlands). Cells were seeded at 1.5×10^6^ cells/well in 6-wells plates and medium was refreshed every other day. After 6 days cells were harvested, washed and resuspended in culture medium. DC purity (CD209 positive, CD14 negative) was checked by flow cytometry.

### Bacterial Co-culture and TLR Transfectants, DC Stimulation

A total of 2×10^5^ DCs were added to 24-wells plates and incubated with either culture medium (RPMI-1640, 2% FBS, 150 µg/ml gentamycin) (negative control), or with *B. breve* or *L. rhamnosus* (all 2×10^6^ microorganisms/well) at a ratio of 10∶1 (bacteria: DC) for 16 hours at 37°C without serum or with the indicated serum fractions. DC phagocytosis was inhibited by first pre-treating DCs with 10 µg/ml cytochalasine D (Sigma Aldrich) for 30 mins at 37°C. In a separate experiment, 1×10^5^ DCs were added to 96 well flat-bottom plates and incubated with either culture medium (RPMI-1640, 1% FBS, 150 µg/ml gentamycin) (negative control), or with *B. breve* or *L. rhamnosus* (all 1×10^6^ microorganisms/well) at a ratio of 10∶1 (bacteria: DC) for 16 hours at 37°C without serum or with the indicated serum fractions. TLR2 activity was inhibited by preincubating cells with 5 µg/ml TLR2 antibodies (clone T2.5, Invivogen, Toulouse, France) or mouse IgG1 isotype control antibodies (clone PPV-06, Bio-Connect, Huissen, The Netherlands) for 30 minutes before the addition of bacteria. 3×10^5^ THP-1 cells/well were seeded in a U-bottom 96-wellsplate and stimulated with the different TLR2 ligands, at the indicated concentrations, in medium or the indicated serum fractions for 16 hours at 37°C. HEK293 TLR2-TLR6 transfectants were seeded in culture medium (DMEM, 10% FBS) at 3×10^4^ cells/well in flat-bottom 96-wells plates and allowed to adhere overnight. Next day cells were washed and co-incubated with *B. breve* at a ratio of 15∶1 (bacteria: cell) with serum-free medium (DMEM, 150 µg/ml gentamycin) or medium with sera at the indicated percentages.

### Bacterial Aggregation and Phagocytosis

To assess bacterial aggregation, 2.5×10^6^ microorganims/well were seeded in flat bottom black 96-wells clear-bottom plates (Corning, Amsterdam, The Netherlands) with serum-free medium (DMEM, 150 µg/ml gentamycin) or medium supplemented with 5% of the different serum fractions for 16 hours. Digital pictures of the wells were obtained using an Olympus SC30 CMOS camera (Olympus, Zoeterwoude, The Netherlands). Images were subsequently processed and analyzed using the public domain program ImageJ, calculating the area covered by objects. To assess bacterial phagocytosis, *B. breve* and *L. rhamnosus* were labeled with the amine-reactive dye pHrodo Red, SE (Life Technologies, Bleiswijk, The Netherlands) in 900 µl of 0.1M sodium bicarbonate, pH 9.2 for 1 hour at room temperature. Labeled bacteria were extensively washed and labeling efficiency was checked using a flow cytometer at pH 4. 2.5×10^6^ Labeled bacteria were co-incubated with 1×10^5^ DCs seeded in U-bottom 96-wells plates in medium (RPMI1640, 150 µg/ml gentamycin) supplemented with the different serum fractions. DCs were collected after 3 hours and analyzed for fluorescence using flow cytometery. DC capacity for phagocytosis was measured using pHrodo Red Dextran 10Kd (Life Technologies, Bleiswijk, The Netherlands). 1×10^5^ DCs were seeded in 96-well flat bottom plates with PBS or PBS supplemented with 5% of the different serum fractions. After 2 hours of incubation, DCs were supplemented with 30 µg/ml pHrodo Red Dextran and incubated for an additional hour after which cells were collected and measured using flow cytometry.

### Cytokine, Luciferase and SEAP Measurements

DC supernatants were analyzed for IL-6 and TNFα (both from R&D Systems, Abingdon, UK) release using ELISA according to the manufacturer’s instructions. HEK293 TLR2-TLR6 transfectants were analyzed for luciferase content via addition of 1 volume of the luciferase substrate: BriteLite (Perkin Elmer, Groningen, The Netherlands) after which Luminescence was measured. THP-1 supernatants were analyzed for secreted embryonic alkaline phosphatase (SEAP) activity using QUANTI-Blue (Invivogen, Toulouse, France) after which OD was measured using a spectrophotometer.

### Statistics

All data was analyzed with GraphPad Prism 4.1. All results are presented as means ± SEM (DC experiments) or means ± SD. Statistical analysis were performed with the use of unpaired two-tailed Students *t*-test where p<0.05 was considered statistical significant.

## Results

### Serum Inhibits Bacterial-induced TLR2/6 Activation

**Figure 1:**
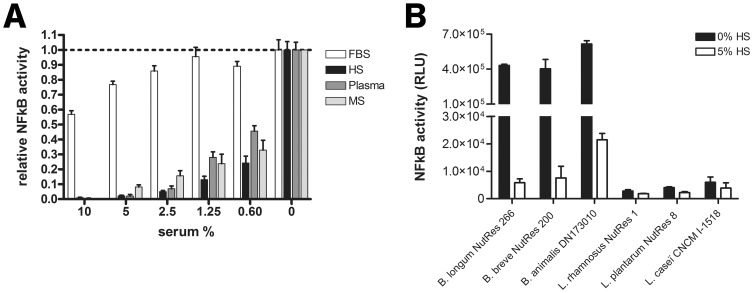
Bacterial induced TLR2/6 activation. TLR2/6 expressing HEK transfectants were co-incubated with different strains of bifidobacteria or lactobacilli. (A) Dose response experiment indicating the effects of the different supplements (FBS = fetal bovine serum, HS = human serum, plasma = human plasma, MS = mouse serum) on TLR2/6 activity. TLR2/6 expressing cells were stimulated with *B. breve* NutRes 200 in a ratio of 15∶1 (bacteria:cell) for 16H. NFκB activity measured as Relative Light Units (RLU) was determined using a Luciferase reporting system as described in materials and methods. Relative NFκB activity was determined calculating the ratios between medium activity and the activity in the different serum fractions. (B) TLR2/6 activity induced by 3 Bifidobacteria and 3 Lactobacilli strains. TLR2/6-expressing cells were stimulated at a ratio of 15∶1 (bacteria:cell) in serum-free or 5% human serum (HS) containing medium. NFκB activity measured as RLU was determined as described above.

To assess the effect of sera on *B. breve* NutRes 200 induced TLR2/6 activation, TLR2 - TLR6 transfected HEK cells were stimulated overnight in the presence or absence of fetal bovine serum (FBS), normal human serum (HS), human plasma (plasma) or mouse serum (MS). Relative to conditions without serum, sera from different species dose-dependently inhibited TLR2/6 activation, with FBS being the least potent (**Figure 1** **a**). To study if this effect was species specific, the human serum capacity to inhibit TLR2/6 activity of three additional *Bifidobacterium* species and three *Lactobacillus* species was determined. In agreement with previous findings [30], *Bifidobacterium* species induced TLR2/6 activity in contrast to *Lactobacillus* species (**Figure 1** **b**). The presence of 5% human serum almost completely abolished TLR2/6 activity of *Bifidobacteria* regardless of species, indicating that human serum does not discriminate between species regarding its inhibitory effect on TLR2 activity. HEK293 TLR null control cells stimulated under similar conditions remained negative for NFκB activation, showing that cellular activation is critically dependent on the presence of the transfected TLR2– TLR6 construct (data not shown).

### HS-EVs Differentially Modulate MAMP-induced TLR2 Responses

**Figure 2:**
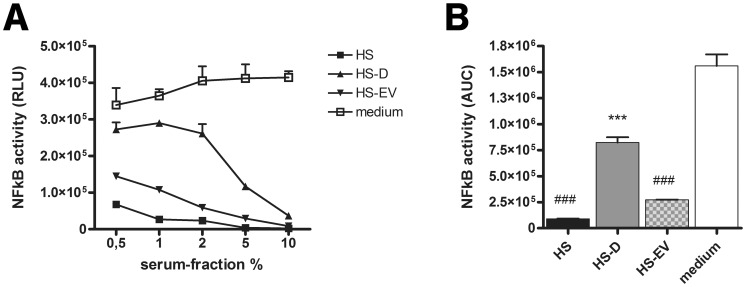
EVs inhibit *Bifidobacterium breve* induced TLR2/6 activation. TLR2/6-expressing HEK transfectants were stimulated with *B. breve* NutRes 200 in a ratio of 1∶15 for 16H. (A) Dose response experiment indicating the effects of the different human serum fractions on TLR2/6 activity (HS = intact human serum, HS-D = EV depleted human serum, HS-EV = human serum EVs in medium). NFκB activity was determined as described in Figure 1. (B) Data presented in Figure 2a were used to calculate Area Under the Curve (AUC) values. HS and HS-EVs dose-dependently inhibited TLR2/6 activity. Hash-signs indicate a significant difference (^##^P<0.01) compared to medium. EV depletion dose dependently rescued TLR2/6 activity. Asterisks indicate a significant difference (^**^P<0.01) compared to HS-D.

**Figure 3:**
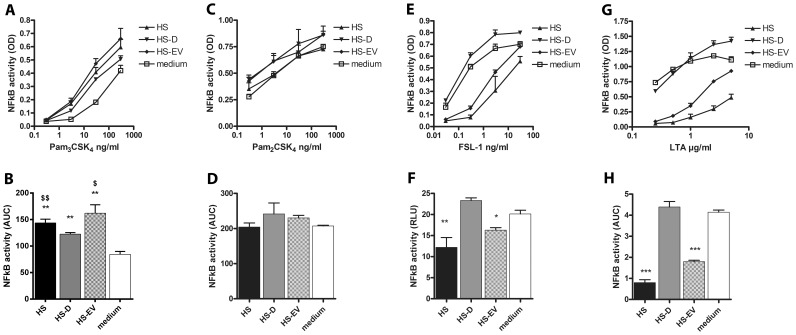
EVs differentially affect synthetic ligand-induced TLR2 activation. THP-1 reporter cells were incubated with synthetic TLR2 ligands addressing different TLR2 heterodimers in serum free medium, or medium supplemented with 5% of the indicated serum fractions (HS = intact human serum, HS-D = EV depleted human serum, HS-EV = human serum EVs in medium). (A, C, E, G) Dose response experiments indicating the effects of respectively Pam_3_CSK_4_, Pam_2_CSK_4_, FSL-1 or LTA stimulation on THP-1 activation. NFκB activity measured as OD values was determined using an alkaline phosphatase reporting system as described in materials and methods. (B, D, F, H) Data presented in Figure 3A,C,E,G were used to calculate Area Under the Curve (AUC) values. (B) Pam_3_CSK_4_ induced TLR2/1 activity was increased in medium supplemented with serum fractions compared to serum-free medium, depletion of EVs reduced TLR2/1 activation compared to HS (^$$^P<0.01) and HS-D (^$^P<0.05). (D) No modulatory effect by serum fractions were observed on Pam_2_SK_4_ stimulation. (E,F) HS and HS-EVs significantly inhibited FSL-1 and LTA induced TLR2/6 activity compared to medium (^***^P<0.001, ^**^P<0.01, ^*^P<0.05).

To specify the inhibitory effect of HS on *B. breve* NutRes 200 induced TLR2/6 activity, HS was depleted for extracellular vesicles (EV). The different fractions, HS, EV-depleted HS (HS-D) and the EV-enriched fraction (HS-EV) were tested for their relative capacity to inhibit TLR2/6 activity (**Figure 2** **a**). Depletion of EVs from HS significantly improved TLR2/6 activity when compared to HS. Medium reconstituted with HS-EVs significantly reduced TLR2/6 activity when compared to HS-D or medium control, suggesting that EVs are the principle factor contributing to the inhibitory capacity of HS (**Figure 2** **b**). Similar results were obtained upon using FBS serum fractions instead of HS (data not shown). TLR2 needs to form heterodimers with TLR1 or TLR6 in order to initiate signal transduction following activation. To research whether the inhibitory effect of HS and HS-EVs affects activation of TLR2/1 and TLR2/6 heterodimers similarly, THP-1 reporter cells were stimulated with the specific ligands Pam_3_CSK_4_ (TLR2/1), Pam_2_CSK_4_ (TLR2) or FSL-1 and LTA (TLR2/6) [31]–[33]. Unexpectedly, addition of HS, HS-D or HS-EV differently affected TLR2 ligand induced THP-1 activation (**Figure 3** **a,c,e,g**). Compared to medium, serum fractions HS, HS-D and HS-EV significantly enhanced THP-1 activation following Pam_3_CSK_4_ ligation. Moreover, depletion of EVs significantly reduced THP-1 activation compared to HS and HS-EVs (**Figure 3** **b**). No differences between HS fractions were measured upon Pam_2_CSK_4_ ligation (**Figure 3** **d**). Both HS and HS-EVs significantly reduced THP-1 activation following FSL-1 and LTA ligation, which could be rescued upon EV depletion (**Figure 3** **f,h**). Additionally, to investigate whether or not other surface TLRs were equally affected, THP-1 reporter cells were stimulated with LPS (TLR4) or flagellin (TLR5). LPS induced NFκB activity was enhanced by all serum fractions in contrast to flagellin induced activity where intact serum but not the serum fractions showed a significant inhibition. Moreover, depletion of EVs significantly reduced LPS induced THP-1 activity compared to HS and HS-EVs (**Figure S1)**. Overall the data show that, depending on the TLR-ligand, EVs display an enhancing effect (Pam_3_CSK_4,_ LPS) or an inhibiting effect (FSL-1, LTA) or no effect (Pam_2_CSK_4_, flagellin). To test whether the effect of EVs is not generically suppressive or enhancive irrespective of TLR signaling, HEK293 TLR null control cells or TLR2/6 transfectants were stimulated with TNFα in the presence of the different HS fractions (data not shown). No differences between TNFα induced NFκB activity could be observed when HS fractions were compared to medium, indicating that EVs selectively modulate TLR induced NFκB activity.

### HS-EVs Inhibition of TLR2/6 Responses is Independent of EV TLR2 Expression

**Figure 4:**
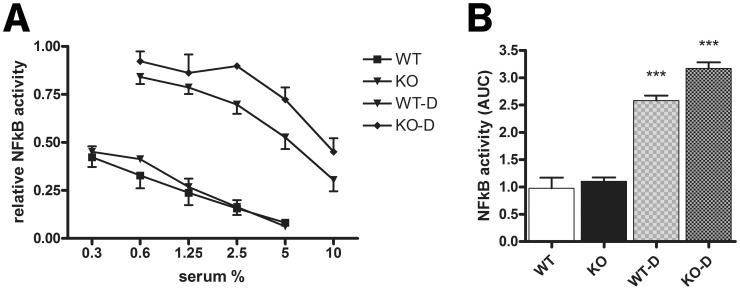
EV-TLR2 expression is not required for the suppressive effect of EVs. TLR2/6 expressing HEK transfectants were stimulated with *B. breve* NutRes 200 in a ratio of 1∶15 for 16H. (A) Dose response experiment indicating the effects of the different mouse serum fractions on TLR2/6 activity (WT = wild type, WT-D = EV depleted wild type serum, KO = TLR2 deficient mouse serum, KO-D = EVs depleted TLR2 deficient mouse serum in medium. NFκB activity was determined as described in Figure 1. Relative activity was determined calculating the ratios between medium activity and the activity in the different serum fractions. (B) Data presented in Figure 4a were used to calculate Area Under the Curve (AUC) values. EV depletion of both WT and KO sera significantly increased TLR2/6 activity (^***^P<0.001).

Proteomic analysis of breast milk derived EVs previously indicated the presence of TLR2 [26]. This finding led us to consider whether TLR2 expression on serum derived EVs might be responsible for the observed inhibitory effect. To that end, HEK TLR2/6 cells were stimulated with *B. breve* NutRes 200 in the presence of serum and EV depleted serum from TLR2^−/−^ deficient (KO) and wild type (WT) mice and their inhibitory effect compared to medium control (**Figure 4** **a**). KO or WT serum was equally effective in inhibiting TLR2/6 activation indicating that EV-TLR2 expression does not play a role in the observed effects. EV depletion of WT as well as KO serum significantly increased TLR2/6 activity (**Figure 4** **b**).

### HS-EVs are Involved in Bacterial Aggregation

**Figure 5:**
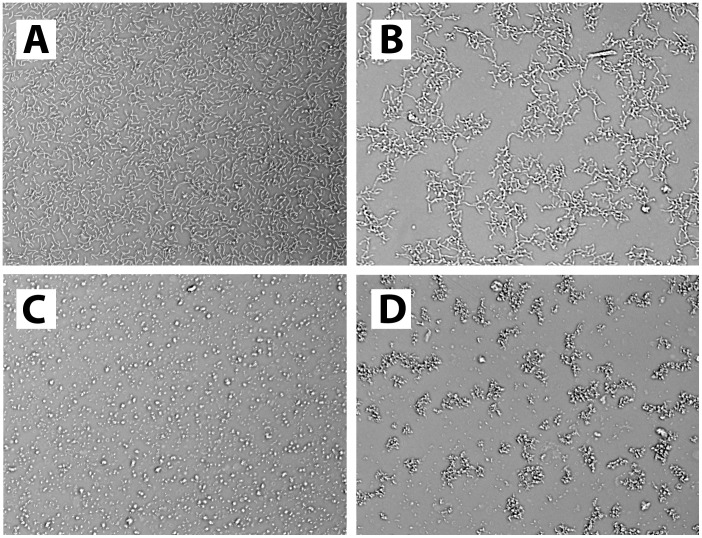
EV mediated bacterial aggregation. Picture A and C respectively represent a typical example of *L. rhamnosus* NutRes 1 and *B. breve* Nutres 200 co-incubated with medium, medium with ExoQuick or medium depleted for EVs. Picture B and D respectively represent a typical example of *L. rhamnosus* NutRes 1 and *B. breve* Nutres 200 co-incubated with HS or HS-EVs.

**Figure 6:**
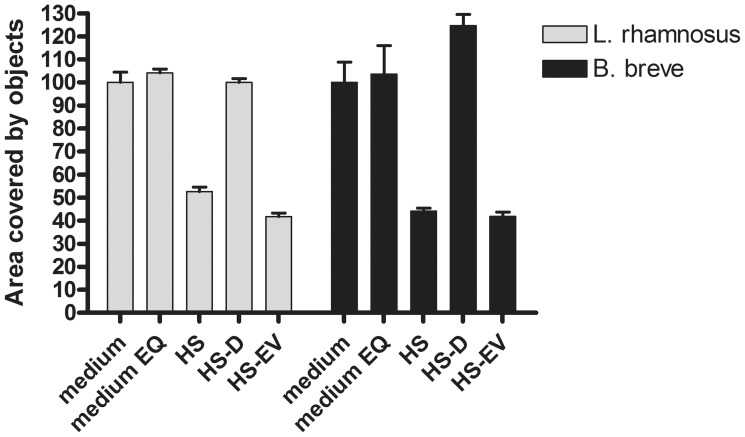
EVs induce bacterial aggregation. *B. breve* NutRes 200 and *L. rhamnosus* NutRes 1 were seeded in flat-bottom 96-wells plates at 2.5×10^6^ bacteria/well in medium, medium with ExoQuick (EQ)(control), HS, HS-D or HS-EV and incubated at 37°C. After 16H cultures were analyzed for aggregation using microscopy and pictures were taken. Pictures were digitally processed and analyzed using ImageJ software, calculating the area covered by objects. Intact serum as well as HS-EVs induced bacterial aggregation, reducing the area covered by objects by approximately 50%. Aggregation was not observed upon EV depletion. No differences between strains could be observed.

The reported presence of PRRs (including but not limited to TLRs) on EVs in combination with the important role of PRRs in host-microbe interactions led us to consider that EVs might interact directly with bacteria which could lead to bacterial aggregation. To that end, pure cultures of *B. breve* NutRes 200, and *L. rhamnosus* NutRes 1 were incubated with serum fractions HS, HS-D, HS-EVs or medium without serum. After overnight incubation cultures were inspected by microscopy. Bacterial aggregation could be detected in cultures supplemented with HS or HS-EVs but not HS-D or plain medium. No differences could be observed in bacterial aggregation between *B. breve* or *L. rhamnosus* (**Figure 5**). Calculation of the relative area covered by objects (see materials and methods) confirms the initial observation, indicating an ∼ 50% decrease in the area covered by bacteria due to bacterial aggregation (**Figure 6**).

### HS-EV are Involved in Bacterial Phagocytosis

**Figure 7:**
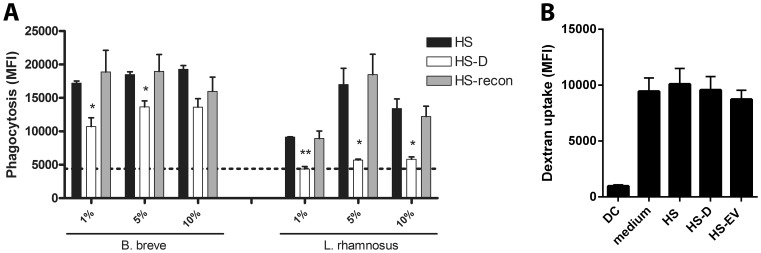
EVs enhance DC phagocytosis of bacteria. 1×10^5^ DCs were co-incubated with 2.5×10^6^ pHrodo-labeled *B. breve* NutRes 200, *L. rhamnosus* NutRes 1 or pHrodo Red Dextran as a control for DC phagocytic capacity at 37°C. (A) Medium was supplemented with increasing concentrations of HS, HS-D or HS-D reconstituted with the serum EV fraction (HS-recon). After 3H, DCs were collected and analyzed for fluorescence using flow cytometery. Degree of phagocytosis is determined as an increase in mean fluorescence index (MFI). EV depletion significantly reduced *B. breve* phagocytosis at 1 and 5% supplementation compared to HS (^*^P<0.05). Phagocytosis of *L. rhamnosus* was reduced to background level (dotted line) upon EV depletion at 1%, 5% and 10% supplementation compared to HS (^**^P<0.01, ^*^P<0.05, ^*^P<0.05, respectively). Reconstitution of HS-D with EVs normalized the phagocytic response compared to HS. Data are represented as mean ± SEM n = 2. Experiment was repeated at least twice with similar results. (B) Medium was supplemented with 5% of the indicated serum fractions and 30 µg/ml pHrodo Red Dextran. After 1 hour DCs were harvested and MFI determined. Medium as well as all serum fractions increased dextran uptake compared to DCs alone (DC). No differences in dextran uptake could be measured between medium and the different serum fractions. Data are represented as mean ± SEM n = 4.

To gain further insight into the relevance of HS-EV interaction with bacteria, we examined whether or not HS-EVs would impact bacterial phagocytosis by DCs. Depletion of EVs from HS resulted in a significantly impaired phagocytosis of both *B. breve* NutRes 200 and *L. rhamnosus* NutRes 1 compared to intact HS. Reconstitution of HS-D with HS-EVs (HS-recon) normalized the phagocytic response when compared to intact HS (**Figure 7** **a**). Depletion of EVs from HS almost completely abolished *L. rhamnosus* NutRes 1 phagocytosis while having a minimal effect on *B. breve* NutRes 200 phagocytosis. To help discriminate effects on DC phagocytic activity from effects on bacterial interactions, DC capacity to phagocytose labeled dextran was measured. No differences in phagocytic capacity could be measured upon supplementation with the different serum fractions indicating that EVs do not interfere with the capacity of DCs to take up particles (**Figure 7** **b**).

### HS-EVs Modulate DC Cytokine Responses Strain-dependently

**Figure 8:**
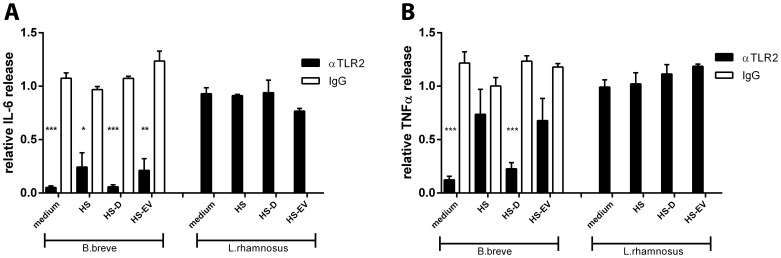
*B. breve* but not *L. rhamnosus* ligate DC-expressed TLR2. 1×10^5^ DCs were co-incubated with 1×10^6^ *B. breve* NutRes 200 or *L. rhamnosus* NutRes 1 at 37°C in medium, HS, HS-D or HS-EVs. TLR2 activity was inhibited by preincubating cells with a specific anti TLR2 antibody or isotype control. After 16H, supernatants were collected and analyzed for IL-6 and TNFα release. Relative cytokine levels were calculated according to the ratio between responses at serum-free medium and serum fraction supplemented medium. (A) Blocking TLR2 significantly inhibited DC IL-6 release after ligation by B. breve but not L. rhamnosus irrespective of the serum fractions. (B) Blocking TLR2 significantly inhibited DC TNFα release after ligation of B. breve in the presence of medium and HS-D but not intact HS or HS-EVs. DC TNFα release in response to ligation of L. rhamnosus was not affected. Data are represented as mean ± SEM n = 4 (^***^P<0.001)(^**^P<0.01)(^*^P<0.05).

**Figure 9:**
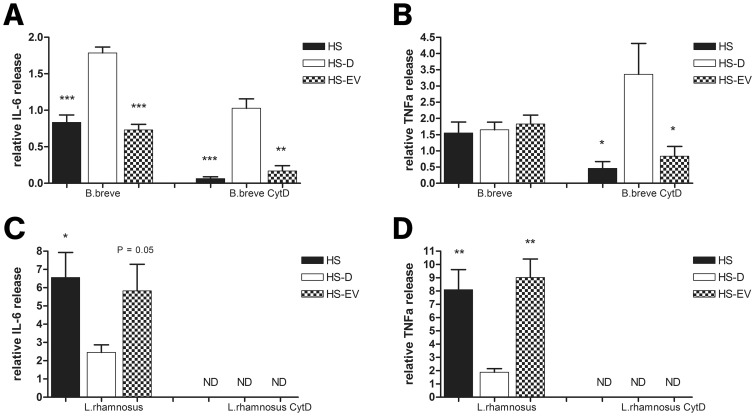
EVs differentially modulate bacterial induced DC cytokine release. 2×10^5^ DCs were co-incubated with 2×10^6^ *B. breve* NutRes 200 or *L. rhamnosus* NutRes 1 at 37°C in medium, HS, HS-D or HS-EVs. After 16H, supernatants were collected and analyzed for IL-6 and TNFα release. In another set of similar experiments, DCs were first pretreated with 10 µg/ml cytochalasine D, blocking bacterial phagocytosis. Relative cytokine levels were calculated according to the ratio between responses at serum-free medium and serum fraction supplemented medium. (A) HS and HS-EVs significantly inhibit *B. Breve* NutRes 200 induced DC IL-6 release compared to HS-D (^***^P<0.001)(^**^P<0.01). TNFα release was not affected but upon blocking phagocytosis a significant different TNFα release between HS, HS-EVs and HS-D could be measured (B) (^*^P<0.05). *L. rhamnosus* NutRes 1 stimulated DCs release significantly more IL-6 (C) and TNFα (D) in the presence of HS or HS-EVs compared to HS-D. Blocking *L. rhamnosus* NutRes 1 phagocytosis inhibited DC IL-6 and TNFα release below detection level (ND). Data are represented as mean ± SEM n = 4.

To further substantiate our findings of HS-EV induced inhibition of TLR2 activity and their contribution to DC phagocytosis, HS-EVs were examined for their effect on DC cytokine release after bacterial challenge. Additionally, to zoom in on the specific effect of HS-EVs on bacterial-induced DC signaling from surface receptors, we performed DC-bacteria co-culture experiments in the presence cytochalasine D (cytD), a known inhibitor of phagocytosis. To establish a role for TLR2 in the interaction of DCs with *B. breve* NutRes 200 and *L. rhamnosus* NutRes 1, DCs were pre-treated with antibodies directed against TLR2 or an isotype control. Blocking TLR2 significantly inhibited *B. breve* induced IL-6 release, independent of the presence of the different serum fractions. TLR2 inhibition had no significant effect on *L. rhamnosus* induced IL-6 release (**Figure 8** **a**). Blocking of TLR2 inhibited *B. breve*-induced TNFα release in the presence of medium or HS-D but not intact serum or HS-EVs, indicating a minor role for TLR2 in the *B. breve* induced TNFα release when incubated in the presence of HS or HS-EVs. TLR2 inhibition had no significant effect on *L. rhamnosus* induced TNFα release (**Figure 8** **b**). Next, the different serum fractions were analyzed for their potential to modulate bacterial induced DC IL-6 and TNFα release. Depletion of EVs from HS (HS-D) significantly increased IL-6, but not TNFα release. Treatment with cytD led to a reduction of both IL-6 and TNFα release, which could be rescued upon EV depletion (**Figure 9** **a,b**). *L. rhamnosus* NutRes 1 exposed DCs release IL-6 and TNFα and depletion of EVs led to a significant reduction in both IL-6 and TNFα cytokines. Treatment with cytD completely abolished DC cytokine production (**Figure 9** **c,d**). Taken together, the data suggests that DC proinflammatory cytokine release upon challenge by *B. breve* is dependent on the TLR2 inhibitory effect of HS-EV in contrast to cytokine release upon challenge by *L. rhamnosus* which is largely dependent on the HS-EV mediated increase in phagocytosis.

## Discussion

Studies on the involvement of TLRs in immune recognition of microbes have helped us to understand how cells, especially DCs and IECs, sense and differentiate between harmless and harmful microbes. However, since microbes share MAMPs, how exactly immune cells differentiate between closely related bacteria is still largely unknown but important in the understanding of immunostimulatory versus immunosuppressive effects of LAB. We and others have previously shown a divergent role for TLR2 in the sensing of bifidobacteria and lactobacilli [17], [30]. Here, we corroborate previous findings demonstrating that specific bifidobacteria, in contrast to lactobacilli, ligate TLR2 and additionally show that TLR2 activation is dependent on the presence of serum. We further show that specifically the EV fraction of serum inhibits TLR2 activation, and that TLR2 activation could be rescued upon EV depletion. Differential effects of the presence of EVs were seen upon analyzing LAB-induced DC proinflammatory cytokine release. Presence of serum enhanced *Lactobacillus rhamnosus*, but not *Bifidobacterium breve*, induced DC proinflammatory cytokine release which was dependent on the presence of EVs. When DC phagocytosis was inhibited, *B. breve* -induced DC IL-6 and TNFα release were impaired in the presence of serum, attributable to the presence of EVs. Blocking *L. rhamnosus* phagocytosis completely inhibited DC cytokine release. This suggests that, in accordance with data on TLR2 inhibition, DC sense *B. breve*, but not *L. rhamnosus*, upon initial contact via membrane-expressed TLR2. Moreover, analogous to the data on HEK TLR2/6 cells, EVs inhibit subsequent *B. breve* induced TLR2 activation. Several types of interaction between EVs and recipient cells are suggested, such as adhesion of EVs to cellular surfaces through ligand-receptor interactions [19]. For instance, blocking antibodies for various integrins, adhesion molecules or tetraspanins significantly reduced DC EV capture [34]. Next to their function in cell-cell and cell-matrix interactions, integrins play a role in phagocytic processes including the clearance of microbes [35]. Additionally, querying a database of molecular data identified in the different subclasses of EVs (Vesiclepedia [36]) indicated the presence of EV-expressed PRRs (i.e. LPS-binding protein, CD14, scavenger receptors) associated with bacterial attachment to host-cells [37]. We therefore hypothesized that EV expression of PRRs (including but not limited to integrins) might mediate microbial attachment. In support of this hypothesis, intestinal epithelium luminal released EVs have been shown to directly bind the surface of *Cryptosporidium parvum* sporozoites which was mediated via unindentified specific molecules on the surface of both *C. parvum* sporozoites and epithelial cells [38]. Our observations on bacterial aggregation suggest that EV indeed interact with LAB and that this interaction results in LAB aggregation, since serum depleted for EVs was not able to do so. Interaction of EVs with bacterial surfaces might additionally expand the repertoire of microbial expressed molecules with host PRR-ligands providing additional molecules for recipient cells to interact with and subsequently influence phagocytic processes. Indeed, our data indicate that phagocytosis of both LAB strains was dependent on the presence of EVs. Interestingly, depletion of EVs only mildly reduced *B. breve* phagocytosis, in contrast to *L. rhamnosus* where phagocytosis was almost completely inhibited. TLRs are not phagocytic receptors by themselves, however, TLR-activation contributes to phagocytic processes [39]. Although we do not provide direct proof, we can hypothesize that the absence of surface TLR triggering by *L. rhamnosus* might be compensated by the EV-interaction with the bacterial surface prompting phagocytic uptake. LAB phagocytosis leads to microbial breakdown and the subsequent intracellular PRR ligation by cell wall products and bacterial DNA which, together with surface TLR2 signaling, contribute to DC cytokine release [17]. The increased DC proinflammatory cytokine response towards *L. rhamnosus* in the presence of EVs can therefore be explained by the EV-mediated increased phagocytosis. The biological relevance of these findings might be two-fold. On the one hand, EV modulation of LAB TLR2 activity and phagocytosis has an impact on the capacity of LAB to modulate subsequent immune responses. On the other hand, EVs in general might contribute to gut immune homeostasis by reducing TLR induced inflammatory responses and increasing microbial clearance [40], [41]. However, whether or not the serum-EV fraction is representative for the EVs present within the intestinal tissue remains to be determined.

Another key point from this study is the differential actions of EVs on specific TLR ligands. Using ligands specifically addressing the different heterodimers of TLR2, TLR4 or TLR5 we made the surprising observation that EVs specifically increase TLR2/1 and TLR4 activation, while having a suppressive effect on TLR2/6 activity or no effect on TLR5 activity. EVs mediate the traffic of a wide variety of lipids, proteins, mRNAs and microRNAs that are important to its biological function [19]. Especially microRNAs have been show to specifically target TLR activity of recipient cells [42]. TLR2 is thought to heterodimerize with TLR1 or TLR6 to broaden the ligand repertoire but upon activation share a similar signal transduction pathway [43]. Since serum and EVs inhibit TLR2/6 activity, while having a stimulatory effect on TLR2/1 and TLR4 activity, it seems unlikely that EV mediated miRNA interference is responsible for the observed effects.

EVs reportedly express TLR2 which might function in analogy to soluble TLR2 (sTLR2) by binding to ligands or via interference on cellular TLR2 heterodimerization ultimately inhibiting subsequent TLR2 activation [26], [44]. However, serum derived from TLR2 knockout animals or wild types was equally effective in reducing TLR2 activation which could be rescued upon EV depletion, ruling out EV-TLR2 expression as a potential mechanism of action.

FSL-1 is similar in molecular make-up to Pam_2_CSK_4_ apart from having a different peptide chain [33], yet our data show that FSL-1 activity is inhibited in contrast to Pam_2_CSK_4_. Similar findings were previously published where activity of a bacterial-derived LP from *Mycobacterium tuberculosis* was found to be suppressed by the presence of serum in contrast to Pam_3_CSK_4_ which is identical in molecular make-up apart from the peptide sequence [45]. In agreement with our data, the effectiveness of serum to inhibit TLR2 responses seems to be dependent on the non-acyl part of the ligands. Although the lipid acyl chains of LPs are critically important for TLR2 heterodimer discrimination and activation [32], the molecular make-up of the peptide chain has been shown to impact TLR2 immunological function [46]. How exactly the peptide moiety of LPs contributes to TLR2 activity is still unclear, but a clue might be derived from the reported interactions of TLR2 with additional PRRs [37]. For instance, CD14 as well as the scavenger receptor CD36 function as TLR2 co-receptor and have been researched in more detail regarding their role in TLR2 biological activity [47], [48]. CD36 does not directly interact with TLR2, but has been reported to bind TLR2 ligands having a negative charge like LTA and FSL-1, in contrast to positively charged ligands like Pam_2_CKS_4_ and Pam_3_CSK_4_ [49]. In contrast to CD36, CD14 directly interacts with the fatty acid portion of triacylated ligands, independent of the peptide moiety [50]. Since EVs are reported to express such PRRs [19], [26], a possible explanation for the reported findings might be that EV-expressed PRRs specifically scavenge LPs, possibly based on their acyl chain make-up or charge of their non-fatty acid part and thereby preventing interaction with surface expressed TLR2. This interdependency has not been researched in detail thus far but a systematically approach in synthesizing bacterial cell wall mimetics with different acyl chains and peptide moieties (LP) or carbohydrate moieties (LTA) would increase understanding of the principles of EV-LP interaction.

TLR activation can be viewed as a double-edged sword. It is critical for host-defense against microbes, but is also linked to inflammatory and autoimmune diseases via their activation by endogenous molecules [15], [51]. TLR responses need to be tightly controlled as prolonged and excessive activation of TLRs can lead to deleterious inflammation and tissue injury detrimental to the host. TLR signaling can be regulated through modification of intracellular pathways following activation, or via interference of TLR ligation by soluble factors and decoy receptors [40], [52]. Although we cannot rule out a possible contribution of miRNA species to the observed effects, our data suggests that the observed effects described are not on the level of host-cell transcription but rather on the level of EV-microbe or EV-specific MAMP interaction which subsequently prevents or enhances host cell surface TLR-activation while at the same time contributes to the clearance of microbes. The biological relevance of EVs enhancing or inhibiting specific TLR responses needs to be addressed in future studies. Determination of the origin of these specific EVs and the receptors involved may lead to new therapeutics for the prevention and/or treatment of inflammatory diseases.

Lastly, potential probiotic strains can be pre-screened *in-vitro* for their immunological potential. Cytokine production following co-culture of LABs with either peripheral blood mononuclear cells or DCs in the presence of FCS allows for ranking of LAB strains according to their pro or anti-inflammatory profile [53]–[55]. However, the value of in-vitro immunoassays as selection criteria for the use of probiotics in human studies remains to be determined [56]. Our data indicate that the predictive value of these screening strategies could be improved by doing experiments in the appropriate serum environment as LAB induced TLR2 activity was only slightly inhibited in the commonly used FCS as compared to mouse or human serum.

In summary, we present experimental evidence highlighting a previous unrecognized role of EVs in modulation of host-microbe responses. Using specific ligands, surface TLR responses were differentially influenced by the presence of EVs. We showed that TLR2 responses were differentially modulated by serum-derived EVs critically important in the response to and recognition of LAB derived cell wall ligands. Additionally, we provide evidence that EVs interact with LAB surfaces and induce bacterial aggregation while at the same time contributing to LAB phagocytosis, mechanisms known to be involved in host-defense responses by facilitation of bacterial clearance. Overall, as EVs can be found in any tissue or bodily fluid, our findings contribute to a better understanding of host-microbe responses important in intestinal homeostasis and unraveling the mechanism of action of probiotics.

## Supporting Information

Figure S1 **EVs differentially effect ligand induced TLR activation.** THP-1 reporter cells were incubated with specific ligands addressing TLR4 and TLR5 in serum free medium, or medium supplemented with 5% of the indicated serum fractions (HS = intact human serum, HS-D = EV depleted human serum, HS-EV = human serum EVs in medium). (A, C) Dose response experiments indicating the effects of respectively LPS or flagellin stimulation on THP-1 activation. NFκB activity measured as OD values was determined using an alkaline phosphatase reporting system as described in materials and methods. Data presented in Figure 3A,C were used to calculate Area Under the Curve (AUC) values. (B) LPS induced TLR4 activity was increased in medium supplemented with serum fractions compared to serum-free medium, depletion of EVs reduced TLR4 activation compared to HS (^$$^P<0.01) and HS-D (^$$$^P<0.001). (D) HS significantly reduced flagellin induced TLR5 activation in contrast to HS-D or HS-EV which showed no effect. Data is represented as mean ± SD (^***^P<0.001, ^**^P<0.01, ^*^P<0.05).
(DOCX)
