# Supplementary material for: Extracellular Vesicles Modulate Host-Microbe Responses by Altering TLR2 Activity and Phagocytosis
Source: PLoS One. 2014 Feb 20;9(2):e89121. doi: 10.1371/journal.pone.0089121 (PMC3930685; doi:10.1371/journal.pone.0089121)
Supplement: Figure S1 — EVs differentially effect ligand induced TLR activation. THP-1 reporter cells were incubated with specific ligands addressing TLR4 and TLR5 in serum free medium, or medium supplemented with 5% of the indicated serum fractions (HS = intact human serum, HS-D = EV depleted human serum, HS-EV = human serum EVs in medium). (A, C) Dose response experiments indicating the effects of respectively LPS or flagellin stimulation on THP-1 activation. NFκB activity measured as OD values was determined using an alkaline phosphatase reporting system as described in materials and methods. Data presented in Figure 3A,C were used to calculate Area Under the Curve (AUC) values. (B) LPS induced TLR4 activity was increased in medium supplemented with serum fractions compared to serum-free medium, depletion of EVs reduced TLR4 activation compared to HS ($$P<0.01) and HS-D ($$$P<0.001). (D) HS significantly reduced flagellin induced TLR5 activation in contrast to HS-D or HS-EV which showed no effect. Data is represented as mean ± SD (***P<0.001, **P<0.01, *P<0.05). (DOCX) [file pone.0089121.s001.docx]

**Supplemental figure S1.** **EVs differentially effect ligand induced TLR activation.**

THP-1 reporter cells were incubated with specific ligands addressing TLR4 and TLR5 in serum free medium, or medium supplemented with 5% of the indicated serum fractions (HS = intact human serum, HS-D = EV depleted human serum, HS-EV = human serum EVs in medium). (A, C) Dose response experiments indicating the effects of respectively LPS or flagellin stimulation on THP-1 activation. NFκB activity measured as OD values was determined using an alkaline phosphatase reporting system as described in materials and methods. Data presented in Figure 3A,C were used to calculate Area Under the Curve (AUC) values. (B) LPS induced TLR4 activity was increased in medium supplemented with serum fractions compared to serum-free medium, depletion of EVs reduced TLR4 activation compared to HS (^$$^P<0.01) and HS-D (^$$$^P<0.001). (D) HS significantly reduced flagellin induced TLR5 activation in contrast to HS-D or HS-EV which showed no effect. Data is represented as mean ± SD (^***^P<0.001, ^**^P<0.01, ^*^P<0.05).
